# Immediate impacts of low-temperature exposure on cardiac autonomic modulation, neuromuscular efficiency, and postural stability in older adults Yangko dancers – a randomized controlled trial

**DOI:** 10.3389/fphys.2026.1785406

**Published:** 2026-04-02

**Authors:** Lijuan Fu, Zhengbin Li, Na Liu

**Affiliations:** College of Human Movement Science, Jilin Sport University, Changchun, Jilin, China

**Keywords:** cold environment, electromyography analysis, heart rate variability, older adults, Yangko dance

## Abstract

**Introduction:**

To investigate the immediate effects of different cold environments on heart rate variability (HRV), surface electromyography (sEMG), and balance in older adults Yangko dance (a traditional Chinese folk dance) participants, and to elucidate the mechanisms underlying increased injury risks during the early exercise phase (10–20 minutes) in cold conditions. This study aims to provide scientific evidence for targeted protective measures.

**Methods:**

A randomized controlled trial was conducted with 120 regular Yangko dancers (age>60 years) from Changchun, China. Participants were stratified into four temperature groups (15 °C, -5 °C, -10 °C, -15 °C; 30 cases in each group) using computer-generated randomization. All participants completed Yangko dance sessions under their assigned temperature conditions. HRV time-domain and frequency-domain indices (SDNN, RMSSD, PNN500, LogLF, LogHF, LF/HF), sEMG signals of the biceps femoris, rectus femoris, and medial gastrocnemius (RMS, IEMG), and balance parameters (RT, MVL, EPE) were measured using heart rate monitors, surface EMG devices, and a balance testing system. The impacts of different cold environments on cardiovascular and motor functions were analyzed.

**Results:**

Compared to 15 °C, the cold groups (-5 °C, -10 °C, -15 °C) showed significant reductions in SDNN, RMSSD, PNN500, LogLF, and LogHF (P<0.05) after the experiment, while LF/HF increased significantly (P<0.05). Progressive hypothermia induced dose-dependent decreases in SDNN (15 °C: 65.4 ± 1.47 vs. -15 °C: 51.21 ± 13.41, P<0.001) and RMSSD, alongside increased LF/HF ratios (P<0.05). sEMG analysis demonstrated temperature-dependent neuromuscular adaptations, with -15 °C exposure eliciting 23.6% RMS elevation but 29.4% IEMG reduction versus controls (P<0.05), indicating enhanced muscle activation but decreased output efficiency. The conclusion of the balance ability test shows that RT is prolonged and DCL is reduced.

**Discussion:**

Cold environments reduce HRV, increase muscle stiffness, and impair balance in the older adults, significantly raise injury risks during the early exercise phase. Recommendations include adequate warm-up, wearing thermal protective gear, and performing joint mobility exercises to enhance exercise safety in cold conditions.

## 1 Introduction

Yangko dance is a traditional folk dance of the Han Chinese in northern China, originating from farming activities. It evolved from farmers’ dances to pray for and celebrate the harvest, gradually developing into a dance form for health promotion of Chinese people (Li et al., 2022). It is assumed to effectively enhance limb coordination, cardiopulmonary function, and overall physical fitness (Lee, 2005, Navari et al., 2024). With the accelerating aging process in Chinese society, the health of the older adults has become a focal point of public concern. Among various approaches to promoting physical and mental health in the older adults, Yangko dance, a traditional fitness activity with profound cultural roots, is favored by older adults due to its moderate intensity and lively format (Li et al., 2022).

Yangko dance is particularly popular in northeastern China, with a large number of participants, especially among the older adults. However, cold winter conditions, pose challenges to Yangko dance participation. Older adults individuals face higher risks of exercise-related injuries in cold environments (Yelkenci et al., 2025). Studies show that muscle elasticity and joint flexibility decrease significantly in cold temperatures, accompanied by reduced reaction time and body coordination (Abaidia et al., 2017). Cold temperatures reduce the fluidity of neuronal cell membranes, impairing the normal opening and closing of ion channels. This slows the conduction speed of nerve impulses, directly compromising the efficiency of signal transmission between the central and peripheral nervous systems (Beker et al., 2018). Studies suggest that in cold environments, the human spleen can contract to release its stored, densely packed red blood cells into the systemic circulation. This process enhances the blood’s oxygen-carrying capacity but also increases its viscosity (Bakovic et al., 2005), which leads to a decrease in cerebral blood flow. These could consequently lead to diminished motor coordination, slower reaction times and a corresponding increase in the risk of falls. Insufficient warm-up and low ambient temperatures often lead to muscle strains, joint sprains, and falls within the first 10–20 minutes of exercise—a high-risk period for injuries during Yangko dance. These injuries not only compromise exercise effectiveness but also pose serious health threats.

Heart rate variability (HRV) is a critical indicator of heartbeat interval fluctuations, which reflect autonomic nervous system activity. Cold exposure is considered as a highly stressful condition that rapidly activates immediate and short-term regulatory mechanisms (Zalewski et al., 2013). During cold exposure, sympathetic nerve-mediated peripheral vasoconstriction and norepinephrine release occur (Johnson et al., 1977), while baroreceptor stimulation in the carotid sinus and aortic arch triggers parasympathetic activation to restore physiological homeostasis (Douzi et al., 2020). However, elevated circulating norepinephrine levels in the older adults can precipitate arrhythmias in congestive heart failure (Kaye et al., 1994, Hausswirth et al., 2013). Cold temperatures increase the risk of junctional rhythms and atrial reentrant arrhythmias. Normal neurological changes reduce myocardial conduction velocity (Mattu et al., 2002), and previous studies report higher incidences of out-of-hospital cardiac arrests during cold weather (Fukuda et al., 2014).

In this study, we focus on investigating the mechanisms underlying cold-induced effects on HRV, muscle function, and balance of older adults Yangko dancers. The study selected four specific temperatures (15 °C, -5 °C, -10 °C, and -15 °C) to reflect typical weather conditions in northeastern China. 15 °C was chosen as a control group, representing the warm conditions that occur during transitional seasons. The other temperatures (-5 °C, -10 °C, and -15 °C) were selected because they are common during sustained outdoor activities in the region’s cold autumn and winter periods. These values allow for observing how various indicators in elderly Yangko dancers change as temperatures gradually drop from mild to moderate cold. These findings will provide scientific evidence for injury prevention and risk reduction in older adults Yangko dancers, enhance exercise safety and offer tailored guidance for cold-weather fitness activities. It will ultimately promote improved physical and mental health in the older adults population. This investigation pioneers the application of multimodal physiological monitoring (HRV, sEMG, posturography) to characterize cold-induced biomechanical adaptations in older adults Yangko dancers.

## 2 Materials and methods

### 2.1 Study design

**Figure 1:**
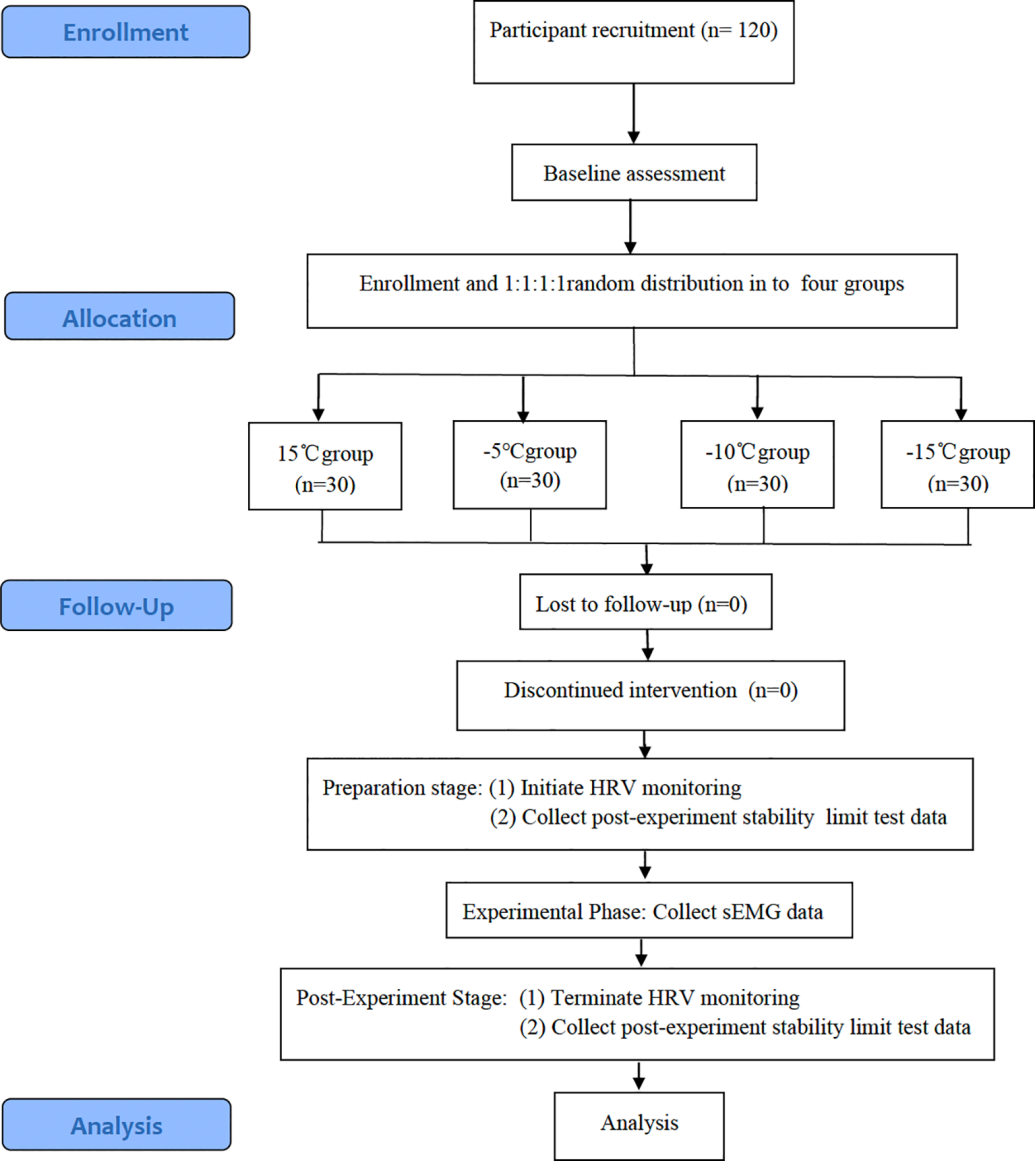
Flow chart of the trial.

This study employed a multi-gradient hypothermia exposure experimental design, and this is a randomized controlled trial with four parallel arms. This study used the single-blind method, in which all subjects were blinded to the temperature conditions, and the assessors who participate in the experiment knew the grouping results but did not know the purpose of this study. A total of 120 eligible older adults participants were randomly assigned to four groups (n=30 per group) using a random number table. Each group was exposed to a distinct low-temperature environment (15 °C, -5 °C, -10 °C, -15 °C), simulated using an environmental chamber (CYPRESS Systems, Model ECT-9000) maintained target temperatures (± 0.3 °C) with 45-50% relative humidity, verified by NIST-traceable thermohygrometers (Testo 635-2) at 1-minute intervals. One hour prior to the experiment, pre-cooling was initiated, maintaining relative humidity at 40%–50%. In order to maintain consistency across experimental conditions, participants were uniformly outfitted in thermal undergarments and lightweight outerwear. In a winter environment, the average clothing insulation value for this elderly population was 2.0 clo (He et al., 2024). Strict protocols were implemented requiring abstinence from strenuous exercise, alcohol consumption, and caffeine intake prior to experimental procedures. In addition, subjects should ensure adequate sleep, avoid high-salt and high-fat foods, and maintain a positive emotional state before the experiment. The protocol began with a 5-minute warm-up phase inside the environmental chamber to acclimate participants to the target temperature. This phase included dynamic stretching and gait activation movements, with heart rate maintained below 100 beats/min. At the target temperature, heart rate should be controlled at 60-70% of the maximum value. The music rhythm was fixed at 120–130 beats per minute and perform 15 minutes of Yangko dance (**Figure 1**).

### 2.2 Participants

120 cases of older adults over 60 years of age who participated in Yangko dance in Changchun were selected as experimental subjects for research and analysis. Prior to the experiment, the participants received relevant training regarding the purpose and procedures of the study. Written informed consent was obtained from all participants prior to their inclusion in the study. This study was conducted in accordance with the ethical principles of the Declaration of Helsinki and was approved by the Academic Ethics Committee of Jilin Sports University (Approval No. 2025010).

### 2.3 Inclusion criteria

(1) Age≥60 years, long-term participation in Yangko dance (≥3 times/week, continuing for more than 1 year); (2) No serious cardiopulmonary disease, osteoarticular disease, or neurological disease; (3) Blood pressure, blood lipid and other basic physiological indicators are in the normal range.

### 2.4 Exclusion criteria

(1) Acute cardiovascular event or sports injury within the last 3 months; (2) Presence of cognitive disorder or inability to cooperate with experimental requirements; (3) Long-term use of medications that affect cardiovascular or neurological function (such as β-blockers); (4) Discomfort or willingness to withdraw during the experiment.

### 2.5 Methods

This study required participants to perform Yangko dance throughout the entire experimental protocol. The data of HRV and sEMG were recorded continuously, covering the complete timeline from the preparatory phase before entering the climate chamber until the end of the experiment upon exiting. Specifically, sEMG indices were extracted during the execution of the key technical movement, i.e. “left-right jumping twists”. Stability limit tests were conducted both before and after the experiment, with a mandatory 1 hour seated rest under room temperature conditions following the pre-test before initiating the cold exposure protocol, and the post-test was conducted immediately after the completion of the cold exposure experiment. To avoid the effects of the diurnal changes in physiology, all experiments were scheduled in the morning. During the data acquisition process, ambient temperature and relative humidity were rigorously controlled to maintain stable conditions, thereby minimizing external disturbances.

#### 2.5.1 Heart rate variability

The R-R interval data were recorded using a Polar H10 heart rate chest strap (manufactured by HUNBEIHAOLE). These data were imported into Kubios Hr-standard 3.4.3 software for the processing of heart rate (HR)-related information. Subsequently, linear analysis (including time domain and frequency domain analysis) was employed to assess the heart rate variability (HRV) indices. The time domain indices encompassed the standard deviation of the mean normal RR intervals (SDNN), the root mean square of the differences between adjacent RR intervals (RMSSD), the percentage of RR intervals with a difference of more than 50 ms (pNN50), as well as low frequency power (LogLF), high frequency power (LogHF), and the LF/HF ratio. In the detection of artifacts (including missed, extra, and misaligned beats) as well as ectopic beats, a medium threshold correction was applied. Artifacts and ectopic beats were identified by comparing each RR interval against a local average interval (0.25 ms). Ectopic beats were corrected by replacing the corresponding RR intervals with interpolated values. Missed beats were addressed by inserting estimated R-wave timings, and extra beats were removed followed by recalculation of the RR interval series. To account for slow linear or more complex trends, non-stationarities in the time series were reduced using a smoothness prior approach (Tarvainen et al., 2014).

#### 2.5.2 Surface myoelectric indices

Measurements were conducted using the Yun Wei 8-wire wireless surface electromyography (WGS-EMG) tester and its associated analysis software. These measurements aimed to detect electromyographic signals in the biceps femoris (BF), rectus femoris (RF), and medial head of the gastrocnemius (MG) muscles of the four groups, both before and after the intervention. Electrode placement adhered to established surface electromyography protocols. For the biceps femoris, electrodes were affixed over the midpoint of the muscle belly, aligned parallel to its fiber orientation. For the rectus femoris, electrodes were positioned at the most prominent portion of the anterior thigh muscle belly, following the longitudinal axis of the muscle fibers. For the medial head of the gastrocnemius, electrodes were placed on the midpoint of the medial muscle belly on the posterior lower leg, oriented perpendicular to the longitudinal axis of the shank. The inter−electrode distance (center−to−center) was consistently set at 20 mm. The reference electrode was placed on a bony surface near the target muscle, such as the midshaft of the anterior tibial crest, ensuring the absence of overlying muscle tissue. Before testing, the skin was meticulously cleaned and sterilized, with the electrode placement area being wiped with medical alcohol to reduce skin resistance. Disposable surface electrodes were utilized to guarantee the precision of signal acquisition. Comprehensive surface electromyography (EMG) signals were recorded and spectrally analyzed using the analysis software to obtain the root mean square (RMS) and integral electromyography (IEMG). The surface EMG data were transmitted in real time to a laptop via Bluetooth and analyzed with Noraxon MR3 software. Raw sEMG signals were initially band−pass filtered (80–250 Hz) using a finite impulse response (FIR) filter within a Lanczos window. The filtered signals were then fully rectified and smoothed using a 50−ms sliding window, and the root mean square (RMS) as well as integrated electromyography (iEMG) values were derived (Konrad, 2006).

#### 2.5.3 Stability limit test

The NeuroCom Balance System was utilized to conduct the Limit of Stability (LOS) test (Koehler-McNicholas et al., 2018) on the subjects. This test encompassed five indices: (1) Reaction Time (RT): Reaction time refers to the time required for the subject to move from the initial position toward the target direction after the system issues a movement command. (2) End-Point Excursion (EPE): End-Point Excursion refers to the straight-line distance between the actual endpoint position reached by the subject’s center of gravity and the target position during the completion of the extreme posture stability test. This metric assesses the accuracy of the subject’s control over the movement of their center of gravity by quantifying the endpoint error of the movement trajectory. (3) Directional Control (DCL): Directional control is defined as the ratio of the actual distance between the center of pressure’s starting position and its endpoint offset position to the shortest distance (a straight line) between these two points. (4) Movement Velocity (MVL): This refers to the average speed at which the center of pressure moves toward a specific target. (5) Maximum Displacement (MXE): Maximum displacement is the greatest displacement of the pressure center from the test starting position toward each target, recorded and described as a percentage. This test quantifies the maximum distance an individual can freely move their center of gravity in a specific direction while standing without moving their feet, taking steps, or losing balance. During the test, participants observed real−time visual feedback of their center−of−mass (COM) position via a cursor on a computer screen and were instructed to voluntarily move their COM toward system−specified targets. At the beginning of each trial, participants were required to maintain their COM within a central target while awaiting combined visual and auditory cues. Upon cue presentation, participants leaned toward the designated target direction, aiming to position their COM as close as possible to the target. Target locations were normalized to individual height, and participants were allotted 8 s to complete the movement. After each trial, participants returned their COM to the central target and waited for the subsequent visual cue.

### 2.6 Sample size estimation

Sample size estimation was conducted *a priori* using G*Power software (version 3.1.9.2, Heinrich Heine University Düsseldorf, Germany) (Faul et al., 2009). Based on preliminary data from 5 participants per group, an effect size of f = 0.379 was derived. For a one-way fixed-effects analysis of variance (ANOVA), with a significance level (α) of 0.05 and a desired power (1 – β) of 0.80, the required total sample size was calculated to be 84 participants (21 per group). This estimation ensures sufficient power to detect the expected group differences at the specified alpha level. Considering the potential dropout rate, a total of 120 cases were included in this study.

### 2.7 Statistical analysis

The data were analyzed using SPSS 23.0 software, with statistical methods including one-way ANOVA and paired t-tests. Between-group comparisons were performed using one-way ANOVA. If the data met the assumption of homogeneity of variance, the LSD test was used to determine significant differences between groups.

## 3 Results

### 3.1 Characteristics of participants

By using a random number table, the subjects were divided into four groups (30 cases in each group) with different temperatures, i.e. 15°C group, -5°C group, -10°C group, -15°C group. There showed no significant differences in participants’ baseline characteristics (P>0.05). (See **Table 1**).

**Table 1:** Comparison of baseline characteristics of participants in each group (Mean ± SD, n=120).

Group	Age	Height (cm)	Weight (kg)	BMI (kg/m^2^)
15°Cgroup	65.4 ± 1.47	167.1 ± 7.05	67.0 ± 7.94	24.0 ± 0.98
-5°Cgroup	65.1 ± 1.63	166.5 ± 7.35	66.9 ± 9.44	24.1 ± 1.28
-10°Cgroup	66.0 ± 1.88	167.4 ± 6.50	67.7 ± 8.02	24.2 ± 1.08
-15°Cgroup	65.7 ± 1.92	166.8 ± 6.83	66.8 ± 7.91	23.9 ± 1.03
*F* value	1.39	0.81	0.77	0.22
*P* value	0.25	0.97	0.97	0.88

### 3.2 Results synthesis

**Figure 2:**
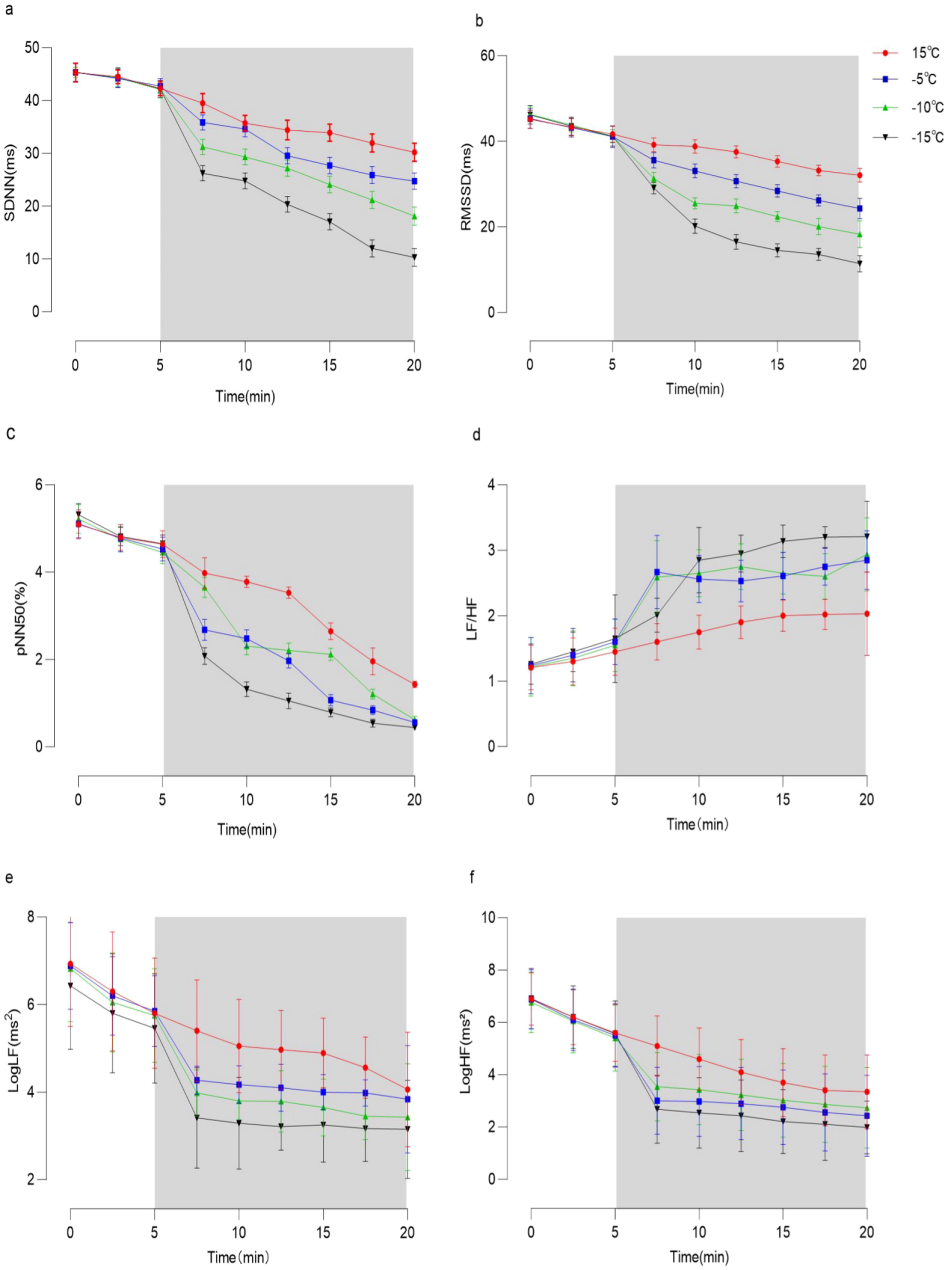
Temporal dynamics of heart rate variability in four experimental groups. **(a)** the trend of standard deviation of the mean normal RR interval (SDNN) over time in 15 °C, -5 °C, -10 °C, and -15 °C environments. The white area in the figure represents the preparation phase before the subject enters the environmental chamber, while the gray area indicates the movement phase of the subject entering the environmental chamber. **(b)** Trend of root mean square deviation (RMSSD) of the period difference between neighboring RRs over time in 15 °C, -5 °C, -10 °C, and -15 °C environments. **(c)** Trend of the percentage of the total number of RR intervals (PNN500) of the neighboring RR intervals with a difference of more than 50ms in 15 °C, -5 °C, -10 °C, and -15 °C environments over time. **(d)** Trend of LF/HF over time in 15 °C, -5 °C, -10 °C, and -15 °C environments. **(e)** Trend of LF power (LogLF) over time in 15 °C, -5 °C, -10 °C, -15 °C environments. **(f)** Trend of HF power (LogHF) over time in 15 °C, -5 °C, -10 °C, and -15 °C environments.

As depicted in **Figure 2**, prior to the experiment, there were no statistically significant differences (P>0.05) among the four groups in terms of SDNN, RMSSD, PNN50, LogLF, LogHF and LF/HF. Compared to 15 °C, the values of SDNN, RMSSD, PNN50, LogLF, and LogHF significantly decreased at lower temperatures (-5 °C, -10 °C and -15 °C), while the value of LF/HF significantly increased after the experiment. With decreasing temperatures, the values of SDNN, RMSSD, LogLF, and LogHF exhibited significant decreases, which showed statistical significance (P<0.05).

As seen from **Table 2**, no significant baseline differences were observed in RMS values of the biceps femoris, rectus femoris, and medial gastrocnemius among the four groups (P > 0.05). The RMS values show a successive downward trend as the temperature decreases after the experiment (P < 0.05).

**Table 2:** Pre-post test RMS comparison across four experimental groups (Mean ± SD, n=120).

Group	BF(μV)		RF(μV)		MG(μV)	
	Pre-test	Post-test	Pre-test	Post-test	Pre-test	Post-test
15°Cgroup	33.94 ± 9.96	37.84 ± 12.32^4)5)^	47.38 ± 16.99	52.43 ± 16.56^4)5)^	37.04 ± 6.57	41.55 ± 6.67^4)5)^
-5°Cgroup	33.78 ± 11.68	43.47 ± 10.35^1)5)^	46.73 ± 21.75	59.23 ± 18.00^1)5)^	38.33 ± 6.02	44.07 ± 6.71^1)5)^
-10°Cgroup	33.62 ± 13.62	47.15 ± 12.44^1)2)^	46.28 ± 23.10	63.00 ± 15.63^1)2)^	38.21 ± 7.84	48.60 ± 8.23^1)2)^
-15°Cgroup	32.30 ± 13.05	51.21 ± 13.41^1)2)3)^	46.14 ± 20.08	70.00 ± 15.56^1)2)3)^	39.28 ± 6.14	51.89 ± 10.12^1)2)3)^
*F* value	0.057	3.260	0.011	2.958	0.281	4.925
*P* value	0.982	0.028	0.998	0.040	0.839	0.004

1) P<0.05 vs. baseline; 2) P<0.05 vs. 15°C group; 3) P<0.05 vs. -5°C group; 4) P<0.05 vs. -10°C group; 5) P<0.05 vs. -15°C group. Abbreviations: BF=Biceps femoris, RF=Rectus Femoris, MG=Medial Gastrocnemius.

The data in **Table 3** showed that there was no statistically significant difference in the IEMG values of the biceps femoris, rectus femoris and medial head of the gastrocnemius muscle among the four groups of subjects before the experiment (P>0.05). After the experiment, the IEMG values of the three muscles mentioned above in 15°C group were significantly higher than those in -5°C group, -10°C group, and -15°C group. The IEMG values were ranked from high to low as 15°C group > -5°C group > -10°C group > -15°C group, and the difference was statistically significant (P<0.05).

**Table 3:** Pre-and post-test IEMG indices across four experimental groups (Mean ± SD, n=120).

Group	BF (μV)		RF (μV)		MG (μV)	
	Pre-test	Post-test	Pre-test	Post-test	Pre-test	Post-test
15°Cgroup	135.30 ± 1.39	129.63 ± 2.86^1)3)4)5)^	176.78 ± 6.61	169.60 ± 7.50^4)5)^	116.59 ± 9.27	110.70 ± 6.91^1)3)4)5)^
-5°Cgroup	134.91 ± 1.00	124.21 ± 2.23^1)2)4)5)^	172.34 ± 9.26	163.91 ± 11.78^1)5)^	115.94 ± 7.36	102.50 ± 5.51^1)2)4)5)^
-10°Cgroup	135.67 ± 1.63	111.42 ± 5.20^1)2)3)5)^	172.74 ± 5.27	159.30 ± 11.13^1)2)^	116.20 ± 9.08	93.76 ± 6.23^1)2)3)^
-15°Cgroup	136.05 ± 1.23	102.10 ± 2.17^1)2)3)4)^	174.73 ± 5.69	153.01 ± 8.20^1)2)3)^	115.66 ± 7.97	91.24 ± 5.72^1)2)3)^
*F* value	2.375	204.214	1.317	7.636	0.028	31.503
*P* value	0.080	<0.001	0.278	<0.001	0.994	<0.001

1) P<0.05 vs. baseline; 2) P<0.05 vs. 15°C group; 3) P<0.05 vs. -5°C group; 4) P<0.05 vs. -10°C group; 5) P<0.05 vs. -15°C group. Abbreviations: BF=Biceps femoris, RF=Rectus Femoris, MG=Medial Gastrocnemius.

**Tables 4**, **5** demonstrated comparable baseline values of RT, MVL, EPE, MXE, and DCL across the four experimental groups (P>0.05). After the intervention, the 15 °C group exhibited significantly less RT but higher MVL, EPE, MXE, and DCL values compared to the other three low temperature groups (-5 °C/-10 °C/-15 °C), which suggesting enhanced equilibrium ability (P<0.05).

**Table 4:** Pre-and post-test RT, MVL and EPE indices across four experimental Groups (Mean ± SD, n=120).

Group	RT (s)		MVL (°/s)		EPE (%)	
	Pre-test	Post-test	Pre-test	Post-test	Pre-test	Post-test
15°Cgroup	0.80 ± 0.33	0.91 ± 0.24^4)5)^	3.71 ± 0.74	3.25 ± 0.58^4)5)^	87.70 ± 9.47	82.52 ± 12.13^4)5)^
-5°Cgroup	0.79 ± 0.24	1.02 ± 0.31^1)5)^	3.65 ± 0.74	2.81 ± 0.81^1)^	86.20 ± 8.47	77.40 ± 9.82^1)5)^
-10°Cgroup	0.81 ± 0.25	1.32 ± 0.18^1)2)^	3.71 ± 0.50	2.64 ± 0.74^1)2)^	86.56 ± 9.00	71.17 ± 13.36^1)2)^
-15°Cgroup	0.81 ± 0.24	1.41 ± 0.09^1)2)3)^	3.75 ± 0.50	2.38 ± 0.76^1)2)^	87.17 ± 6.81	67.78 ± 13.05^1)2)3)^
*F* value	0.020	17.392	0.061	3.759	0.910	4.352
*P* value	0.996	<0.001	0.980	0.016	0.965	0.008

1) P<0.05 vs. baseline; 2) P<0.05 vs. 15°C group; 3) P<0.05 vs. -5°C group; 4) P<0.05 vs. -10°C group; 5) P<0.05 vs. -15°C group. Abbreviations: RT=Reaction Time, MVL=Movement Velocity, EPE=Endpoint Movement.

**Table 5:** Pre-and post-test MXE and DCL indices across four experimental groups (Mean ± SD, n=120).

Group	MXE (%)		DCL (%)	
	Pre-test	Post-test	Pre-test	Post-test
15°Cgroup	78.56 ± 16.66	71.70 ± 10.41^1)4)5)^	79.33 ± 10.21	72.00 ± 7.86^1)5)^
-5°Cgroup	76.36 ± 15.52	66.66 ± 11.05^1)5)^	78.50 ± 10.10	68.83 ± 8.39^1)5)^
-10°Cgroup	75.99 ± 13.10	61.52 ± 8.76^1)2)^	76.70 ± 10.10	64.23 ± 13.85^1)^
-15°Cgroup	77.36 ± 15.51	58.21 ± 13.53^1)2)3)^	76.23 ± 10.60	61.86 ± 10.54^1)2)3)^
*F* value	0.086	4.277	0.307	2.856
*P* value	0.967	0.009	0.820	0.045

1) P<0.05 vs. baseline; 2) P<0.05 vs. 15°C group; 3) P<0.05 vs. -5°C group; 4) P<0.05 vs. -10°C group; 5) P<0.05 vs. -15°C group. Abbreviations: MXE= Maximum Excursion, DCL= Directional Control.

**Figure 3:**
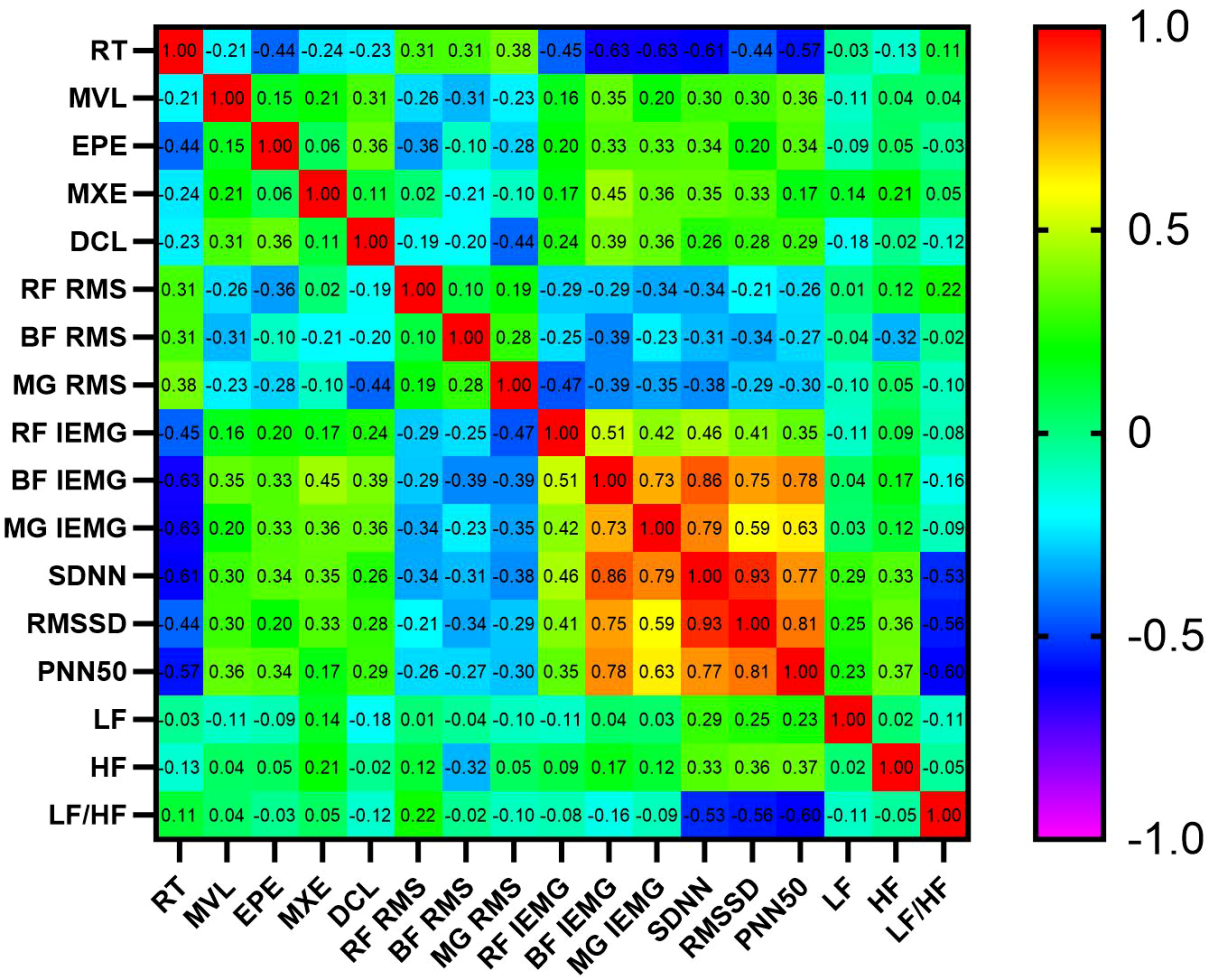
Pearson correlation heatmap for dataset variables.

Pearson correlation analysis (see **Figure 3**) revealed significant associations among HRV, sEMG and balance ability indicators. Between HRV and balance parameters, SDNN showed a significant negative correlation with RT (r = −0.61, P<0.01), which could imply that optimization of autonomic nervous function might effectively shorten reaction time and enhance balance control efficiency. Regarding HRV and sEMG indices, SDNN exhibited a very strong positive correlation with BF IEMG (r = 0.86, P<0.01), suggesting that increased HRV may be closely associated with improved neuromuscular recruitment levels in lower limb muscle groups. For sEMG parameters and balance outcomes, MG IEMG was significantly negatively correlated with RT (r = −0.63, P<0.01), indicating a potential link whereby elevated sEMG activity in lower limb muscles could contribute to enhanced balance performance.

Based on the results of Pearson correlation analysis, indicators with statistically significant differences were screened, and further regression analysis was conducted on these highly correlated indicators to examine their predictive validity. The results of the linear regression analysis presented in **Tables 6**, **7** demonstrate that the constructed regression model exhibits good predictive efficacy for reaction time (RT). The model yielded a multiple correlation coefficient of R = 0.677, a coefficient of determination of R² = 0.458, and an adjusted R² = 0.449, indicating that the temperature-group coding explains approximately 44.9% of the variance in RT. The overall model significance test was highly significant (F(1, 58) = 49.082, p < 0.001), confirming a reliable overall fit of the regression equation. The predictive effect of temperature-group coding on RT was significant (B = 0.179, p < 0.001). That is, when controlling for other variables, each one-unit increase in temperature-group coding was associated with an average increase of 0.179 units in the predicted RT.

**Table 6:** Linear regression model predicting post-intervention reaction time.

Regression model	R	R²	Adjusted R²	F-value	P-value	SEE
RT=0.722 + 0.179×temperature	0.677^a^	0.458	0.449	49.082	0.001	0.221

^a^Dependent variable: RT.

**Table 7:** Excluded variables in the regression model.

Model	Variable	In beta	T-value	P-value	Partial correlation	VIF
1	MG IEMG	-0.226b	-1.392	0.169	-0.181	2.88
1	PNN50	-0.116b	-0.759	0.451	-0.1	2.473

b Predictors: (Constant), temperature.

## 4 Discussion

### 4.1 Effect of temperature on heart rate variability in older adults performing Yangko dance

Changes in HRV indices could effectively reflect this dynamic adjustment of the autonomic nervous system. By extracting and analyzing cardiovascular control information contained in HRV signals, it is possible to quantitatively assess the balance between cardiac sympathetic and vagal nerves.

The results indicate that as the temperature decreased, the heart rate variability parameters such as SDNN, RMSSD, LogLF, and LogHF all showed a significant reduction. This may be attributed to the fact that exposure to a cold environment could inhibit the activity of both the sympathetic and parasympathetic nervous systems in the elderly individuals. Zhu et al. (Zhu et al., 2019) found the similar results which were observed in the experiment of transitioning from a normal temperature environment to a -10 °C low-temperature environment. Previous study has shown that HRV indices decreases with age (Nicolini et al., 2012), the skin vasoconstriction response in the elderly is weakened, and the sensitivity of their skin vasomotor response to sympathetic nerve stimulation is reduced. Furthermore, the increase in metabolic rate induced by cold is also attenuated in the elderly. Animal experiments have demonstrated that the basal time-domain HRV in aged mice is significantly lower than that in young mice in a cold environment, with SDNN values approximately 65% lower (Axsom et al., 2020), indicating that aging can lead to a decline in autonomic nerve regulation function.

The LF/HF ratio is highly sensitive to changes in environmental temperature and human thermal sensation. Huang et al. found that during cold exposure, the body activates compensatory mechanisms by stimulating the sympathetic nervous system (SNS) to maintain core body temperature and reduce heat loss (Huang et al., 2011). SNS activation enhances thermogenesis through vasoconstriction, shivering thermogenesis, and elevated metabolic rates. This study shows that under low-temperature conditions (-5 °C, -10 °C, and -15 °C), the LF/HF ratio exhibits an increasing trend compared to that at 15 °C. Specifically, the decrease in LF is smaller than that in HF, resulting in a higher ratio. The underlying mechanism may involve the sympathetic nervous system playing a dominant role in the autonomic response to cold environments, which helps maintain body temperature through energy conservation and reduced heat loss.

### 4.2 Effect of temperature on RMS and IEMG Indices in older adults performing Yangko dance

In this study, the sEMG signal characteristics of older adult Yangko dancers in hypothermic environments were consistent with a pattern that could be described as “high activation, low efficiency”, aligning with observations by Chaillou et al. (Chaillou et al., 2022). Specifically, the RMS values of the gastrocnemius, rectus femoris and biceps femoris muscles exhibited a marked increase at lower temperatures, it could be interpreted as an enhanced neural drive required to sustain muscle contraction under cold conditions. Conversely, the decrease in the IEMG value suggests a potential reduction in mechanical output efficiency per unit of muscle activation (Gao et al., 2025). The co-occurrence of elevated RMS and diminished IEMG may reflect an inefficient neuromuscular adaptation, wherein greater neural excitation does not appear to translate proportionally into mechanical work. It is plausible that in short-term tasks, this inefficiency could predispose individuals to rapid exhaustion, while in the long term, it might contribute to muscle atrophy or compensatory postural disorders.

Based on the above interpretation, the observed “high activation, low efficiency” state could be understood as a compensatory response, where muscles may counteract cold-induced stiffness through heightened neural recruitment, albeit at the cost of excessive energy consumption and diminished mechanical output. If this interpretation stands, such an adaptation might accelerate fatigue accumulation, potentially explaining the increased mobility challenges and injury risk observed in aging populations exposed to cold conditions.

### 4.3 Effect of temperature on RT, MVL, EPE, MXE and DCL indices in older adults performing Yangko dance

The data show that low-temperature environments significantly influence key metrics of postural control, including reaction time (RT), movement velocity (MVL), end-point excursion (EPE), maximum excursion (MXE), and directional control (DCL). Specifically, the observed increase in RT, coupled with reductions in MVL, EPE, MXE, and DCL, is consistent with a measurable decline in dynamic balance performance under cold conditions.

The lengthened RT could plausibly be attributed to cold-induced alterations in sensorimotor processing, such as a potential reduction in nerve conduction velocity or diminished sensitivity of muscle spindles, which would contribute to a lag in the postural feedback loop (Chaillou et al., 2022). This delay in initiating corrective movements may elevate the risk of falls, particularly in populations with inherently slower reaction times, such as older adults. The diminished DCL values may suggest an impairment in the precision of voluntary movement. One possible explanation, drawn from the existing physiological literature, is that low temperatures may delay calcium ion release from the sarcoplasmic reticulum and decrease the activation efficiency of fast-twitch muscle fibers (Type II), leading to insufficient muscle contraction speed and higher failure rates in dynamic tasks such as emergency stops and turns (Chaillou et al., 2022). Additionally, previous studies suggest that low temperatures impair the synergy between the vestibular, visual, and proprioceptive systems, resulting in diminished multitasking ability, reduced proprioceptive input accuracy, and compromised central integration (Arkkukangas, 2023). We hypothesize that when the center of gravity shifts beyond physiological compensation limits, dynamic balance control becomes unstable, probably increasing the risk of ankle sprains or compensatory lumbar strain; furthermore, joint stiffness forces muscles to maintain posture through isometric contractions, accelerating ATP depletion and lactic acid accumulation, which induces early fatigue and ultimately compromises dynamic balance capability.

### 4.4 Integrated effects of low temperature on multidimensional physiological indicators and their association with fall risk

Exposure to low temperatures may elevate the risk of falls by disrupting autonomic nervous system function, neuromuscular regulation, and postural control. Our findings demonstrate that, compared to the 15 °C control group, participants in the -5 °C, -10 °C, and -15 °C groups exhibited significant reductions in SDNN, RMSSD, PNN50, LogLF, and LogHF, alongside a marked increase in the LF/HF ratio. These HRV alterations suggest that low temperatures suppress parasympathetic activity and compromise autonomic balance. Surface electromyography (sEMG) analysis further revealed temperature-induced neuromuscular adaptations: the -15 °C group showed a 23.6% increase in RMS amplitude (p<0.05) and a 29.4% decrease in IEMG (p<0.05) compared to controls, indicating enhanced muscle activation but diminished contraction efficiency. This observation aligns with previous work by Guidi et al., which reported aberrant HRV patterns under muscle fatigue (Guidi et al., 2017). We therefore speculate that the HRV abnormalities observed in the present study may partially reflect underlying neuromuscular regulatory changes, although this mechanism was not directly tested. Balance assessments confirmed that cold exposure prolonged reaction time and reduced the dynamic control limit (DCL), potentially impairing balance regulation.

According to Dorey et al., HRV serves as a key indicator of autonomic nervous function, and abnormal alterations in HRV are directly associated with fall risk. Specifically, as age increases, overall cardiac autonomic regulation declines (Dorey et al., 2021), with resting RMSSD showing a significant negative correlation with the number of falls, and the standing LF/HF ratio showing a significant positive correlation. This suggests a reduction in the inhibitory regulation and flexibility of parasympathetic control over heart rate (Suh, 2023). Tekin et al. further noted that HRV is significantly positively correlated with neuromuscular coordination (Tekin et al., 2025), and a severe decline in proprioceptive function, which can further impair balance regulation, may exacerbate this association. Integrating these findings with our results, we speculate that stressors such as low temperatures may induce additional neuromuscular adaptive changes, potentially driving the increase in fall risk (Germano et al., 2016). The study by Razjoyan et al. also confirms that HRV can serve as an objective monitoring indicator for fall risk (Razjouyan et al., 2017).

Collectively, our data indicate that low-temperature exposure is significantly associated with HRV abnormalities, diminished neuromuscular efficiency, and compromised balance capacity. These correlated changes suggest a plausible pathophysiological pathway whereby autonomic dysfunction, potentially interacting with impaired neuromuscular control, contributes to an elevated risk of falls. However, the causal relationships within this proposed pathway require further validation through studies that directly intervene on specific autonomic or neuromuscular functions.

## 5 Limitations

The findings of this study provide valuable practical insights for elderly individuals engaging in physical activity in cold climates. However, as the participants are all long-term Yangko dancers, the generalizability of the results to the broader elderly population or to those involved in different forms of physical activity may be limited. Ansdell et al. (Ansdell et al., 2020) suggested that the response of physio-logical systems to exercise differs between males and females, potentially mediating the beneficial effects in healthy and clinical populations. But we didn’t consider the gender differences in this work, customized recovery plans that improve performance and health outcomes for both sexes could be informed by research on the physiological reactions to cold exposure in relation to sex in subsequent research. Furthermore, the literature’s reports of COVID-19’s effects on exercise pathophysiology have raised significant questions regarding the utility of cryogenic therapies in patient rehabilitation plans (Baratto et al., 2021). Examining how cryotherapy affects workout performance and recuperation in this situation may yield insightful information and consent to personalize the treatment (Pastorino et al., 2021) in the future.

Furthermore, a fundamental limitation lies in the inferential nature of the proposed mechanistic pathways. While our interpretations provide a coherent framework linking the observed data to established physiological principles, they remain hypothetical and were not directly tested within the current study design. This highlights the critical need for future research to experimentally validate these specific causal pathways using targeted interventions.

Nonetheless, the present study is primarily centered on investigating the immediate effects of varying low-temperature conditions on physiological indicators during Yangko dance performance among elderly individuals and elucidating the associated underlying mechanisms, whereas the delineation of a safe exercise range for this specific population was not incorporated into the current study design. As temperatures decrease, the characteristic defect in equilibrium function primarily manifests as an increase in RT. However, our experimental design employed non-linear temperature gradients; subsequent research should therefore adopt a linear temperature design to accurately determine the cutoff value for low-temperature environmental conditions affecting RT.

## 6 Conclusion

A low-temperature environment significantly elevates the risk of sports injuries for the older adults during the early stages of exercise (10 to 20 minutes). Under such conditions, the range of motion in their joints decreases markedly, leading to a corresponding weakening of balance ability and deterioration of body stability. This results in an increased tendency to sway and lose balance during movement. Additionally, gait stability is compromised, and the coordination of lower limb joints becomes abnormal, further increasing the likelihood of falls. Low temperatures also tend to reduce heart rate variability among the older adults, impair autonomic nervous system function, and induce early-onset muscle fatigue, which collectively hinder the body’s ability to provide effective support and protection during physical activity. These factors interact synergistically, rendering the older adults more susceptible to injuries such as muscle strains, joint sprains, and falls in cold environments.

## Data Availability

The original contributions presented in the study are included in the article/supplementary material. Further inquiries can be directed to the corresponding author.
